# Preoperative Preparation and Anesthesia for Trabeculectomy

**DOI:** 10.5005/jp-journals-10008-1198

**Published:** 2016-05-12

**Authors:** Tom Eke

**Affiliations:** Consultant, Department of Ophthalmology, Norfolk and Norwich University Hospital, Norwich, UK

**Keywords:** Anesthesia, Intracameral anesthesia, Local anesthesia, Peribulbar anesthesia, Retrobulbar anesthesia, Subconjunctival anesthesia, Sub-Tenon’s anesthesia, Topical anesthesia, Trabeculectomy.

## Abstract

Preoperative preparation should improve the likelihood of successful trabeculectomy surgery. The team can reconsider the appropriateness of the proposed surgery, and steps can be taken to maximize the chance of a good outcome. For example, adjustments to anti-hypertensive or anti-coagulant medications may be made, and topical ocular medications adjusted.

Choice of anesthesia technique is of particular relevance to the trabeculectomy patient. Some anesthesia techniques are more likely to have serious complications, and glaucoma patients may be at higher risk of some sight-threatening complications, because the optic nerve is already damaged and vulnerable. Posterior placement of local anesthesia (retrobulbar, peribulbar, posterior sub-Tenon’s techniques) could potentially damage the optic nerve, and thereby cause “wipe-out” of vision. Anesthesia technique may influence the likelihood of vitreous bulge and surgical difficulty. Regarding long-term control of intraocular pressure, there is no good evidence to indicate that any particular anesthesia technique is better than another. There is little high-quality evidence on this topic. The author’s preferred technique for trabeculectomy is subconjunctival-intracameral anesthesia without sedation.

**How to cite this article:** Eke T. Preoperative Preparation and Anesthesia for Trabeculectomy. J Curr Glaucoma Pract 2016; 10(1):21-35.

## PREOPERATIVE PREPARATION: DOES THE PATIENT REALLY NEED A TRABECULECTOMY

When considering trabeculectomy surgery, one should of course consider the alternatives. Any surgery carries the risk of severe complications, which may be sight-threatening or even life-threatening. Anesthesia can also be risky, with risk of sight-threatening complications of local anesthesia (LA) and life-threatening complications with LA and also with general anesthesia (GA). In addition to the immediate-term risks of surgery and anesthesia, one should consider the longer-term risks of trabeculectomy surgery, such as worsening cataract, hypotony, bleb leakage and early or late endophthalmitis. Undergoing a trabeculectomy demands a good deal of cooperation from the patient, with multiple eye-drops in the short-term, and regular follow-up visits. These considerations should be weighed against the eye’s risk of developing visual loss from glaucoma, the individual’s general health and circumstances, and numerous other factors when deciding what might be best for this patient. Often, the best option will be not to do a trabeculectomy, but instead manage the patient in a different way. This review will aid in the decision-making process, and should help the clinician and patient to choose a procedure which has the best balance of safety, efficacy, and likelihood of lifelong vision for the patient. For further discussion, see previous reviews of anesthesia for glaucoma surgery.^1,2^

### Issues of the “Only Eye”

Eye surgeons are fortunate in that their patients usually have two eyes, and it is possible to manage reasonably well with just one eye. Thus, a sight-threatening complication in one eye may not necessarily have a huge impact on the patient’s overall visual function―they still have the “spare eye”. For a patient who has only one seeing eye (the “only eye”), any loss of vision would definitely affect their well-being in a major way. Therefore, the “only eye” must be looked after very carefully. This presents a paradox for the ophthalmologist: Glaucoma in an “only eye” may need aggressive management in order to minimize the likelihood of future visual loss, but any surgical complication could be devastating.

The glaucoma specialist should also consider the reason why the patient has vision in one eye only. If vision was lost in the first eye from a complication of trabeculectomy, both patient and surgeon may wish to avoid similar surgery to the second eye. If vision was lost to glaucoma, then the decision-making process may be more straightforward.

When considering whether or not to operate, the risks and benefits of surgery must be discussed fully with the patient, in order to agree a strategy. This discussion is even more important for a one-eyed patient, because of the potential for devastating sight loss, should a severe complication occur. Depending on circumstances, the best option in many cases may be to avoid surgery.

**Table 1:** **Table 1:** Preoperative medication changes to consider for your trabeculectomy patient

*Medication*		*Anesthesia-related concerns*		*Trabeculectomy-related concerns*		*Actions to consider*	
Antiplatelet, anticoagulant medication		May increase risk of bleeding from LA (Table 3)		Risk of bleeding during or after surgery		Choice of LA technique Consider stopping these medications before/after surgery Liaise with the physician who prescribed this medication	
Glaucoma drops				Prolonged preoperative use may increase risk of failure, particularly with preserved eye drops (“activated conjunctiva”)		Consider reducing number of drops preoperatively (e.g. acetazolamide, non-preserved drops) Consider anti-inflammatory drops preoperatively	
Anti-infectives		Risk of orbital infection, especially, with posterior sub-Tenon’s LA		Endophthalmitis risk		Preoperative povidone iodine drops 5% is proven to minimize risk preoperative antibiotic drops not proven to minimize risk	

If surgery is felt to be necessary, then all practicable steps should be taken to maximize the likelihood of success and to minimize the likelihood of harm (Table 1). This principle should, of course, be applied to all eyes of all patients.

### Preoperative Medication: Should Glaucoma Medications be Discontinued, and When?

The question of preoperative adjustment of glaucoma medications has not been fully resolved. It is recognized that an “activated conjunctiva” will reduce the likelihood of trabeculectomy success, because the conjunctival scarring reaction has already been “activated” by an insult, such as prior surgery to the conjunctiva, or long-term glaucoma drops. Older eye drops, such as adrenaline and dipivefrin appear to have a large effect, any drop preserved with benzalkonium chloride (BAC) appears to have a moderate effect, and preservative-free drops appear to have a minimal effect, though the evidence base remains weak.^3^ To maximize the likelihood of trabeculectomy success, many glaucoma surgeons will reduce the number of eye drops and/or change to preservative-free medication, in the weeks prior to surgery.^4,5^

### Preoperative Medication: Additional Medication to Improve Success Rate

Most trabeculectomy patients will have been using topical glaucoma medications for some time, and this can result in an “activated conjunctiva”, as described above. There is some evidence to show that a 1 month course of fluorometholone^4^ or preservative-free indomethacin eye drops^5^ will improve the state of the conjunctiva preoperatively. It is assumed that this should improve the likelihood of trabeculectomy success, though the evidence base is still weak.^3^

### Other Aspects of Preoperative Assessment: Ocular

Various ocular problems might increase the likelihood of trabeculectomy failure or complications. Risk factors should modified as appropriate, e.g., blepharitis should be treated (e.g., with a course of an oral tetracycline,^3^ in order to minimize the risk of infection and over-healing. A highly myopic eye will be at higher risk of globe perforation from an LA needle: It is preferable to avoid peribulbar or retrobulbar LA in these eyes. Ocular assessment, combined with a general health assessment, will assist surgical planning: Who should do the operation, and what adjustments should be made to maximize the likelihood of success?

### Other Aspects of Preoperative Assessment: Non-ocular

All surgical patients should have a preoperative assessment of their general health, to include screening for risk factors that could be adjusted before surgery. Ideally, this should be done a few weeks prior to the anticipated date of surgery, to allow time for any preoperative adjustments to be made.^6^ Taking a medical history may disclose a condition which means that trabeculectomy might be at high risk of complication (e.g., severe coagulation disorder) or unnecessary (e.g., a terminal illness). High blood pressure and anticoagulant/antiplatelet medications may influence the likelihood of severe choroidal hemorrhage (see later): These are modifiable risk factors so they should be identified, and acted upon, as early as possible. A history of medication allergies and intolerances should be sought, e.g., if a patient is allergic to latex, or the antibiotic you normally use, this will give time to organize an alternative. A preoperative assessment will allow planning for the type of anesthesia to be used (LA or GA). Further advice on preoperative assessment, and other aspects of the operative process, is available in the 2012 National Guideline on Local Anesthesia for Ophthalmic Surgery.^6^

### Preoperative Medication: Minimizing Risk of Postoperative Endophthalmitis

Endophthalmitis is a feared complication of any surgery. Intraocular infection can result in a blind/painful eye, so all efforts should be made to minimize this risk. Preoperative use of povidone-iodine eye drops has been shown to reduce the risk of endophthalmitis.^7^ Preoperative antibiotic prophylaxis has not been shown to give any additional reduction in risk, and in many countries they are not used routinely.^6,8^ Use of intracameral or subconjunctival antibiotic at the end of surgery, and postoperative antibiotic drops, are standard practice and will be discussed elsewhere.

### Anesthesia Options for Trabeculectomy

Trabeculectomy can be performed using GA, or any of the LA techniques. Detailed instructions for each LA technique can be found in textbooks of ophthalmic anesthesia.^9^ International surveys have shown that all techniques are in use for trabeculectomy: General anesthesia, sharp-needle blocks with retrobulbar anesthesia (RBA) or peribulbar anesthesia (PBA), sub-Tenon anesthesia (STA) blocks using a blunt cannula, sub-conjunctival anesthesia (SCA) with sharp needle, topical anesthesia (TA) or topical-intracameral anesthesia (ICA). Some surgeons routinely use a combination of one or more of these LA techniques.^10^ Anesthesia techniques for trabeculectomy can be categorized as follows:

- Local anesthetic behind the globe: Retrobulbar, peribulbar, posterior sub-Tenon’s LA (LA can be given by surgeon or anesthetist, with or without sedation).
  – *Retrobulbar:* Sharp needle injection of LA, aiming inside the cone of the rectus muscles.
  – *Peribulbar:* Sharp needle injection of LA, aiming outside the cone of the rectus muscles.
  – *Posterior sub-Tenon:* Blunt-cannula injection in the sub-Tenon’s space, aiming at or behind the equator.
- Local anesthetic to the front part of the globe only: Anterior sub-Tenon’s, sub-conjunctival, topical, intra-cameral LA (normally given by the surgeon as part of the procedure, can be given with or without sedation).
  – *Anterior sub-Tenon:* Blunt-cannula injection in the sub-Tenon’s space, aiming in front of the equator.
  – *Sub-conjunctival:* Sharp-needle injection of LA to the sub-conjunctival space (some publications will describe this as “anterior sub-Tenon LA”).
  – *Topical LA*: Eye drops or gel LA to anesthetize conjunctiva (and deeper layers, with special technique)
  – *Intracameral LA:* Non-preserved LA irrigated into the anterior chamber during surgery.
- General anesthesia: This can only be given by an anesthetist.

The main advantages and disadvantages of each anesthesia technique are summarised in Table 2. The main agents used in LA are summarized in Table 3. The main complications are discussed below, and summarized in Table 4.

### Factors Influencing Choice of Anesthesia for Trabeculectomy

Various factors should be considered when choosing the anesthesia technique for a trabeculectomy. The main ones are as follows:

- Risks of complications from the LA or GA
  – Sight-threatening complications
  – Life-threatening complications
  – “Wipe-out”.
- Operating conditions
  – Ease of surgery
  – Likelihood of long-term intraocular pressure (IOP) control
  – Likelihood of surgical complications.
- Patient factors
  – General health
  – Life expectancy
  – Medications
  – Expectations and anxiety
  – Ability to position for surgery.
- Surgeon factors
  – Preference, technique
  – Personality
  – Level of training
  – Surgeon speed.
- Ocular/orbital factors
  – Globe size and shape
  – Orbit, lids, etc.

### Sight-threatening Complications of LA: Globe Perforation

Sight-threatening complications are more likely to occur with sharp needle LA (retrobulbar or peribulbar), particularly if technique is poor.^11^ Potential problems include needle perforation of the globe, which could cause scleral laceration, vitreous hemorrhage and retinal detachment. Longer (myopic) eyes are at higher risk, because they are longer and wider, and more myopic eyes are more likely to also have a posterior staphyloma.^12^

**Table 2:** **Table 2:** Anesthesia options for trabeculectomy, with main advantages and disadvantages

*Technique*		*Main advantages-any surgery*		*Main* *disadvantages-any surgery*		*Main advantages-trabeculectomy*		*Main* *disadvantages-trabeculectomy*		*Special* *considerations for trabeculectomy*	
General anesthesia		Patient asleep and unaware May be the only option for uncooperative patient		Time Expense Personnel Needs hospital facilities Life-threatening complications		Good operating conditions; no chemosis or hemorrhage		Stay suture needed		Avoid systemic hypotension (ischemia may worsen visual field defect) Avoid postoperative nausea and vomiting (may cause choroidal hemorrhage)	
Retrobulbar		Good analgesia and akinesia		Sight-threatening complications Globe perforation needle damage to optic nerve retrobulbar hemorrhage “wipe-out”, etc. Life-threatening complications brainstem anesthesia		Good operating conditions		Stay suture needed Risk of “wipe-out” May cause subconjunctival hemorrhage and chemosis May cause bulgy eye		Care with LA mixture (Table 3) Ensure intraorbital pressure is back to normal before commencing surgery (but avoid over-use of compression)	
Peribulbar		Good analgesia and akinesia		Sight-threatening complications Globe perforation needle damage to optic nerve retrobulbar hemorrhage “wipe-out”, etc Life-threatening complications brainstem anesthesia		Good operating conditions		Stay suture needed Risk of “wipe-out” May cause subconjunctival hemorrhage and chemosis May cause bulgy eye		Care with LA mixture (Table 3) Ensure intraorbital pressure is back to normal before commencing surgery (but avoid over-use of compression)	
Posterior sub-Tenon’s with blunt cannula (e.g., via inferonasal snip)		Good analgesia and akinesia Lower risk of sight-threatening or life-threatening complications, compared to peribulbar, retrobulbar		Sight-threatening complications Globe perforation needle damage to optic nerve retrobulbar hemorrhage “wipe-out” etc. Life-threatening complications brainstem anesthesia		Good operating conditions		Stay suture needed Risk of “wipe-out” More likely to cause subconjunctival hemorrhage and chemosis		Care with LA mixture (Table 3) Ensure intraorbital pressure is back to normal before commencing surgery (but avoid over-use of compression)	
Anterior sub-Tenon’s with blunt cannula (e.g., by surgeon, during surgery)		“No risk” of life-threatening or sight-threatening complications Good analgesia		Potentially mobile eye		Good operating conditions No stay suture needed “Mobile eye” makes surgery quicker, easier Surgeon can give LA during surgery (start with topical)		Potentially mobile eye, need to ensure patient can cooperate subconjunctival hemorrhage and chemosis		In literature, some subconjunctival LA injections by surgeon are described as “anterior sub-Tenon’s”. Some surgeons prefer to use a stay suture, to stop eye movement Could convert to posterior sub-Tenon’s “on the table”, if needed	
Subconjunctival		“No risk” of life-threatening or sight-threatening complications Good analgesia		Potentially mobile eye		Good operating conditions No stay suture needed “Mobile eye” makes surgery quicker, easier Surgeon can give LA during surgery (start with topical)		Potentially mobile eye, need to ensure patient can cooperate subconjunctival hemorrhage and chemosis Previously thought to be risk factor for bleb failure or leaky bleb		Best to give LA under operating microscope, to avoid vessels and minimize risk of hemorrhage or globe perforation Some surgeons prefer to use a stay suture, to stop eye movement Could convert to posterior sub-Tenon’s “on the table”, if needed Previous concerns about bleb failure or leaky bleb appear to be refuted by recent evidence	
Topical and intracameral		“No risk” of life-threatening or sight-threatening complications		Potentially mobile eye Needs careful technique for good analgesia (e.g., time for LA to work, use gel LA or sponges or cocaine drops)		Good operating conditions No stay suture needed “Mobile eye” makes surgery quicker, easier		Higher risk of patient discomfort? Potentially mobile eye, need to ensure patient can cooperate Special drops/gel may not be readily available		Some surgeons prefer to use a stay suture, to stop eye movement Could convert to posterior sub-Tenon’s “on the table”, if needed	

**Table 3:** **Table 3:** Main options for inclusion in LA mixture, with main advantages and disadvantages

*Agent in LA* *mixture*		*Main advantages-any surgery*		*Main* *disadvantages-any surgery*		*Main advantages-trabeculectomy*		*Main* *disadvantages-trabeculectomy*		*Special* *considerations for trabeculectomy*	
Lidocaine (lignocaine)		Good safety record Good analgesia				Good analgesia Good akinesia (depending on technique)				Sufficient for most trabeculectomies with any LA technique	
Bupivacaine (marcaine)		Good safety record Good analgesia				Longer duration action than lidocaine				Longer duration of action, suitable for prolonged operations	
Hyaluronidase		Encourages spread of LA in orbit: Quicker onset of akinesia Minimizes risk of postoperative diplopia?		Animal derived (from sheep or cow) Rarely, swollen orbit after surgery (sight-threatening) Human recombinant version expensive, unavailable in many countries				Risk of vision loss from orbitopathy		Glaucoma eyes may be at higher risk of blindness, if hyaluronidase orbitopathy occurs	
Adrenaline (epinephrine)		Vasoconstrictor, previously thought to improve block quality		Vasoconstrictor, could cause vision loss (nerve or retina infarct)				Risk of “wipe-out”		Best avoided for glaucoma patients	

**Table 4:** **Table 4:** Main major complications of LA, and summary of immediate management (see guideline on LA for ophthalmic surgery^6^)

*Complication*		*LA technique that could cause this complication*		*Symptoms and signs*		*When it can present*		*Immediate action*		*Other* *comments*		*Special* *considerations for trabeculectomy*	
Brainstem anesthesia		Retrobulbar peribulbar (rarely) posterior sub-Tenon’s		Impaired consciousness or coma Apnea Cardiovascular instability Fitting		During LA injection or within minutes		Stop injecting Get help Start resuscitation Transfer to intensive care unit		You need to be prepared for this possibility, if using this LA technique. Need resuscitation equipment, training, personnel			
Optic nerve damage from LA		Retrobulbar peribulbar (rarely) posterior sub-Tenon’s		Vision does not improve after surgery		After surgery		Look for alternative causes (e.g., infarct)				See section on wipe-out	
Globe perforation		Retrobulbar peribulbar (rarely) sub-Tenon’s (rarely) sub-conjunctival		Pain during injection Bleed into eye (hyphema) Choroidal hemorrhage Retinal detachment		During LA injection (pain, eyeball hard, globe may “pop” if intraocular injection) Surgeon may see hyphema or reduced red-reflex 50% are not noticed until post-operatively		Stop injecting! Look for signs of perforation Refer to vitreo-retinal surgeon		Myopes at higher risk because of big/wide globe and posterior staphyloma. If injection LA is needed, a single medial peribulbar injection is safer		If subconjunctival LA, give injection using operating microscope, to minimize risk	
Retrobulbar hemorrhage		Retrobulbar peribulbar (rarely) Posterior sub-Tenon’s		Tense orbit hemorrhage in orbit subconjunctival hemorrhage (bleeding may not be visible at first)		During LA injection Within minutes of LA injection (rarely, later)		If severe, decompress orbit with lateral canthotomy and cantholysis Defer surgery		Slight increase in risk for patients on anticoagulant or antiplatelet medication		Orbital bleed may increase risk of bulgy eye/choroidal hemorrhage Subconjunctival bleed may increase risk of bleb failure	
Surgical difficulty: poor patient cooperation		Any LA technique, especially if patient does not know what to expect		Patient overanxious and/or unable to stay still and cooperate		Usually becomes evident early in surgery		Discuss with patient: e.g., they may want to be re-positioned or may need more LA. Consider sedation or GA instead		Preoperative explanation and counseling will minimize risk of this problem Preoperative assessment should identify patients who are likely to need sedation or GA		Poor cooperation is usually evident early in surgery, but most patients can be reassured. If you think you may need to abandon surgery and reschedule with GA or sedation, try to do this before entering the anterior chamber	
										Offer regular “wriggle breaks” for patient comfort. Hand- holding by an assistant will reassure the patient		Risk of hypotony and bleeding while awaiting completion of surgery	
Surgical difficulty: “bulgy eye” (positive vitreous pressure)		Retrobulbar peribulbar posterior sub-Tenon’s		Anterior chamber keeps shallowing		During surgery Before surgery (tense orbit)		Reposition patient in a more head-up position (reduced hemostatic backpressure if eye is “at the top”) Anterior chamber maintainer, or viscoelastic to anterior chamber Tighter sutures		Pressure to the orbit after the LA will reduce this risk (e.g., manual compression or Honan balloon) Consider supra-choriodal hemorrhage as an alternative cause		Glaucoma patients are at risk of wipe-out from high orbital pressure, particularly if prolonged	
Surgical difficulty: mobile eye		Anterior sub- Tenon’s sub- conjunctival topical- intracameral		Patient cannot keep eye still		Before surgery During surgery		Check that anesthesia is adequate, top up as needed Corneal stay suture Consider changing to different LA (e.g., posterior sub-Tenon’s)				Some surgeons routinely use a corneal stay suture with these anterior LA techniques	

Many trabeculectomy surgeons prefer to use a sharp-needle LA, despite the risk of globe perforation and other LA complications. Risk may be reduced by using short needles, having the globe in the primary postion (looking straight ahead) for the LA injection, injecting in one site only, and choosing either a medial (nasal)^13^ or far interotemporal site for injection.^11^ Globe perforation will usually require urgent management by a vitreoretinal surgeon.

### Sight-threatening Complications of LA: Retrobulbar Hemorrhage

If the LA needle damages an artery, this may cause an arterial bleed into the orbit. This may cause globe ischemia and blindness. Eyes with glaucoma may be at higher risk of developing blindness if a retrobulbar hemorrhage occurs. The optic nerve is already damaged and may be more susceptible to ischemic insult. A tense orbit from retrobulbar hemorrhage will usually require urgent decompression. Simple techniques for decompressing the orbit with lateral canthotomy/cantholysis have been described.^14,15^

### Sight-threatening Complications of LA: Needle Damage to Optic Nerve

An inadvertent needle injury to the optic nerve could also cause partial or complete blindness.^11,16^ Needle injury to the optic nerve could also cause death, because an injection of LA along the optic nerve sheath may cause brainstem anesthesia.

### Sight-threatening Complications of LA: Hyaluronidase Orbitopathy

Many ophthalmologists will add hyaluronidase to their LA mixture, to aid the diffusion of the LA through the orbit. This can give quicker onset of good operating conditions.^17^ In most countries, the hyaluronidase is derived from an animal source (usually sheep). Occasionally, a severe inflammatory reaction may occur, with a hot swollen orbit worsening over a few days. This inflammation may increase orbital pressure: Patients with glaucoma appear to be at higher risk of losing vision, should hyaluronidase orbitopathy occur.^18,19^

Risk of hylauronidase orbitopathy can be minimized via the following strategies:

- *Avoid using hyaluronidase for LA blocks:* This may mean slower onset of good operating conditions, and higher likelihood of postoperative diplopia.
- *Use low dose of hyaluronidase in LA blocks:* Current recommendation is for 15 International Units per milliliter of LA mixture (most commonly, a vial of hyaluronidase contains 1,500 International Units)
- *Use human-derived hyaluronidase for LA blocks:* In recent years, hyaluronidase from a human recom-binant deoxyribonucleic acid (DNA) source has been available in some countries (e.g., USA, where it is marketed as Hylenex®): This appears to have a greatly reduced risk of hylauronidase orbitopathy
- *Use an anterior LA technique (e.g., topical/subcon-junctival):* These LA techniques are not improved by hyaluronidase.

### Sight-threatening Complications of LA: Impaired Optic Nerve Head Blood Flow

If an anesthetic is placed behind the globe (retrobulbar, peribulbar, posterior sub-Tenon’s LA), then this may impair blood flow to the optic nerve.^20-22^ The effect is thought to be due to the large volume of LA in the orbit interfering with normal blood flow, with contributions from pressure-lowering devices (e.g., Honan balloon), pharmacological effects of LA agents, and/or vasoconstrictors in the LA mixture. A reduction in optic nerve blood flow could potentially result in worsening of visual field, or even “wipe out” of all vision from the eye (see next section). Eyes with glaucoma may be at particular risk of this problem, because the optic nerve has already been damaged by glaucoma, and may be more vulnerable to ischemic insult.

Vasoconstrictors (e.g., adrenaline/epinephrine) may exacerbate ischemia, and may be more likely to cause loss of visual field in glaucoma patients. Numerous studies have shown a reduction in pulsatile ocular blood flow with LA blocks, which is more marked if adrenaline/ epinephrine is included in the LA mixture.^23,24^ Blood flow is unaffected when there is anterior placement of LA (e.g., topical/subconjunctival).^20^ Anecdotal evidence indicates that epinephrine is indeed a risk factor for visual loss. A hospital that had a high rate of visual loss after LA blocks found that the problem was solved by omitting epinephrine from the LA mixture.^25^

### Sight-threatening Complications of LA and GA: “Wipe-out” of Visual Field

Glaucoma patients are at risk of “wipe-out” of the visual field, also referred to as “snuff syndrome”.^1,2,26,27^ This term is used to describe visual loss after surgery in glaucoma patients, without any obvious cause. A “partial wipe-out” may manifest itself as an unexplained worsening of visual field after surgery. Sharp-needle LA could potentially cause “wipe-out”, without any obvious signs of the sight-threatening complications described above.^16^ Patients with glaucoma have an optic nerve which is already damaged, which may be more susceptible to further damage if there is increased orbital pressure or ischemic insult.^2^

Injecting LA behind the Globe could cause “wipe-out” via the following mechanisms:

- Direct trauma to the optic nerve, from LA needle or metal cannula
- Trauma to arteries serving optic nerve or nerve head, from LA needle or metal cannula
- Needle trauma causing a small hematoma that presses on the optic nerve.
- Impaired optic nerve head blood flow (see section above). The increase in orbital pressure from a large volume of injectate may compress the optic nerve, particularly if the injectate becomes localized to one of the potential anatomical ‘compartments’ of the orbit.
- Prolonged pressure to the orbit with a Honan balloon or other pressure device
- Adrenaline (epinephrine) is a potent vasoconstrictor: Anecdotal reports indicate that it may indeed be a cause of “wipe-out”, and many glaucoma surgeons prefer to avoid the use of adrenaline in the LA mixture.

Thus, any LA behind the eye (RBA, PBA, STA) could potentially cause “wipe-out”. General anesthesia could also cause wipe-out via other mechanisms, e.g., systemic hypotension causing an optic nerve head infarct. To minimize the risk of wipe-out, it is advisable to use an anterior LA technique (subconjunctival LA, anterior sub-Tenon’s LA, topical-intracameral LA).

### Life-threatening Complications of LA: Brainstem Anesthesia

If the LA needle can damage the optic nerve directly, a ‘needle in optic nerve’ could also cause death.^28^ If the LA is inadvertently injected under the coverings of the optic nerve, it will track back to the brainstem. Neurological effects of brainstem anesthesia include impaired consciousness or coma, apnea, cardiovascular instability and even death. With proper support (e.g., urgent transfer to an intensive therapy unit), patients can be expected to recover without permanent damage.^28^ This complication has also been described with posterior sub-Tenon’s LA.^66^ Clinicians should be aware of this possibility, and should be prepared to resuscitate a patient, if required.^6^

### Life-threatening Complications of LA and GA: Anaphylaxis

It is extremely rare for patients to have an anaphylactic reaction to a local anesthetic.^6^ Anaphylactic reactions to hyaluronidase appear to be rarer than the delayed “hyaluronidase orbitopathy” described above. Anaphylaxis may also occur during GA.

### Life-threatening Complications of GA

Numerous other life-threatening complications may occur during GA. For further details, see any textbook of anesthesia.

In glaucoma surgery, the anesthetist should be aware of issues specific to the glaucoma patient. Eye drops used to treat glaucoma will be absorbed systemically. The anesthetist should be aware if the patient has received beta-blockers, alpha-adrenoceptor agonists, muscarinics, carbonic anhydrase inhibitor, osmotic diuretics, etc. Also, if topical medications, such as atropine or acetylcholine are used during surgery, the anesthetist should be informed.^2^

Historically, ecothiopate (Phospholine Iodide®, an acetylcholinesterase inhibitor) eye drops were a significant concern with GA. If suxamethonium anesthesia were used, the cholinesterase in these eye drops could cause prolonged paralysis (‘scoline apnea’). This medication is no longer available in many countries.

### Other Sight-threatening Complications of LA and GA: Difficult Surgery

Each of the above anesthesia techniques has its advantages and disadvantages, and none is suitable for 100% of cases. Common concerns are listed as follows:

*Anxiety and mobile eye:* Some patients are so anxious that they are unable to tolerate any type of LA. An anxious and mobile patient means that surgery will be difficult, with a high risk of surgical complications (these patients will require GA, or a different approach to their glaucoma management).

*LA block and bulgy or immobile eye:* Using a “LA behind the globe” technique will minimize the risk of complications from uncontrolled eye movements, but some surgeons find the operating conditions imperfect. A large volume of LA behind the eye may cause positive vitreous pressure or “bulgy eye” which can cause iris prolapse and other surgical difficulties. The immobile eye means that either a corneal stay suture or superior rectus suture is needed, in order to expose the surgical area. These sutures can cause significant problems, e.g., a superior rectus suture could cause significant hemorrhage, and corneal stay sutures can be placed too deep (flat anterior chamber), or too superficial (cutting out). A corneal stay suture may pull on the trabeculectomy flap, particularly if there is a bulgy eye: This can make it difficult to get the tension right when suturing the trabeculectomy flap and conjunctiva, and over-drainage may result.

*Topical/subconjunctival anesthesia and mobile eye:* Some surgeons are happy to use topical LA for the majority of cases, but this does require a reasonable degree of patient cooperation―the patient should be able to “look down” and keep the eye still, in order to expose the surgical area. Again, there is the potential risk of complications from uncontrolled eye movements. Some surgeons will use a corneal stay suture, to keep the eye immobile and in downgaze. The author prefers topical/subconjunctival LA, for a population of patients who almost all speak English. In over 12 years of trabeculectomy, we have had no complications that could be attributed to eye movement.

*Eccentric eye position with GA or orbital LA block:* With GA, the eye is usually immobile but can sometimes adopt an “eccentric” position-often in upgaze,^29^ particularly if the patient is only lightly anesthetized. This problem may also occur when LA is given behind the eye. The globe may adopt a position of upgaze or lateral gaze. Again, a stay suture is usually necessary.

### Sight-threatening Complications of LA: Choroidal/Expulsive Hemorrhage

Choroidal hemorrhage during or after surgery is a rare but potentially devastating cause of sight loss. It remains a much-feared complication of eye surgery.^30,31^ Historically, this was more of a concern during large-incision cataract surgery, because the large wound provided a route for the lens, vitreous and even retina to be expelled from the wound: an “expulsive hemorrhage”. With small incision surgery (e.g., trabeculectomy, phacoemulsification), choroidal hemorrhages should not turn into expulsive hemorrhage, but they can still cause severe sight loss.

There are several strategies to minimize the risk of choroidal hemorrhage. Much of the evidence on risk comes from case-control studies: Old age, high preoperative IOP, systemic hypertension, cardiovascular problems, anticoagulants, elevated episcleral venous pressure, myopia, aphakia or prior vitrectomy are associated with increased risk of choroidal hemorrhage.^30-33^ It is logical to control as many of these risk factors as possible, prior to trabeculectomy. However, there have been no good controlled studies to look at whether controlling any of these risk factors might actually decrease the incidence of severe bleeding.

Glaucoma surgeons have several options to minimize the risk of choroidal hemorrhage. These include: pre-operative management of antiplatelet/anticoagulant medications, preoperative blood pressure and IOP management, patient positioning, surgical options and anesthesia options.^30,31^

Regarding anticoagulant or antiplatelet therapy (e.g. warfarin/coumadin, aspirin), many glaucoma specialists will discontinue these medicines prior to surgery.^34,35^ This is because of the perceived risk of a sight-threatening hemorrhage, during or after surgery. Conversely, many glaucoma surgeons will ask their patients to continue these medications throughout the peroperative period.^36-38^ Reasons for continuing these medications might be the risk of life-threatening thrombosis if medications are stopped, or the lack of strong evidence of risk to sight. If considering stopping these medications in the peroperative period, the surgeon should discuss this with the physicians who prescribed this medication.^6^ If discontinuing these medications, common practice is to resume treatment 1 or 2 weeks postoperatively, provided there is no hypotony. Some glaucoma specialists may choose a different surgical option for these patients, preferring non-penetrating surgery or cycloablation instead of trabeculectomy. The expert committee of ophthalmologists and anesthetists that produced the 2012 Guideline on LA for ophthalmic surgery concluded that: “For more complex [non-cataract] surgery with a higher risk of bleeding, anticoagulants and antiplatelet agents may compromise surgical outcome and influence the choice of anesthesia. Currently, there is insufficient evidence to make anesthesia-specific recommendations regarding continuation or cessation of these drugs. Each patient and procedure should be treated on its own merits with multi-disciplinary input as required”.^6^

Anesthesia technique may influence the likelihood of choroidal hemorrhage, though again there is little by way of concrete evidence. Periocular LA (retrobulbar, peribulbar, posterior sub-Tenon’s) will transiently increase the intraorbital pressure,^39,40^ and may reduce venous return from the eye, thereby increasing the likelihood of a peroperative bleed. Thus, surgeons should wait until the orbital pressure has normalized before commencing surgery. If using GA, the patient should be not be too ‘lightly’ anesthetized, because bucking and straining (Valsalva) may cause a bleed during surgery. Likewise, postoperative retching/vomiting after GA may have a similar effect. Proponents of “anterior” LA (topical, sub-conjunctival, anterior sub-Tenon’s) cite the lack of effect on retrobulbar blood flow,^20^ though some have suggested that the “uncontrolled” eye movement may increase the risk of a bleed. In practice, this does not appear to be a significant concern.

Patient position may influence the likelihood of peroperative choroidal hemorrhage. In the head-down (Trendelenburg) position, orbital and intraocular pressure becomes elevated, and pressure is decreased in the more upright position.^41-44^ Thus, a head-up position should reduce the likelihood of choroidal hemorrhage. Obesity is increasingly common. If an obese patient is made to adopt the supine (flat) position for surgery, this will also raise the orbital pressure, because of venous pressure from abdominal and other fat that is at higher “altitude” than the globe. Therefore, it is preferable to have an operating chair or table that allows LA patients to sit up a little, so that the eye is “at the top”. This is particularly important for obese patients.^45^ In practice, most patients do prefer to sit up a little for LA eye surgery.^46^

Another strategy to minimize the risk of choroidal hemorrhage is to reduce a high IOP prior to surgery. Again, there is no strong evidence to suggest exactly what level of IOP is a “dangerous” IOP that should be reduced prior to commencing trabeculectomy. Of course, other risk factors (listed above) should also be considered. In practice, many surgeons will have a “cut-off IOP”, above which IOP lowering is considered, which may typically be between 30 and 40 mm Hg. Options to reduce IOP include eye drops, or systemic osmotic agents, such as acetazolamide, glycerol or mannitol. Intravenous mannitol is generally felt to be best at giving rapid IOP lowering, and many surgeons use it to reduce high IOP preoperatively. Osmotic agents have the disadvantage of drawing fluid into the vascular compartment, which means that the patient will then produce more urine. A full bladder may lead to patient discomfort and agitation, which may then cause surgical difficulties if LA is used. Mannitol has other potential disadvantages, including hypotension, electrolyte imbalance, and pulmonary edema.^47,48^

Systemic blood pressure (BP) is another issue: Hypertension is associated with risk of choroidal hemorrhage and other complications.^30-33^ Case-control studies of choroidal hemorrhage have shown an association with higher peroperative BP and/or pulse rate.^31,49,50^ However, some studies indicated that “taking cardiovascular medication is a better predictor of [choroidal hemorrhage] than systemic hypertension and elevated systolic BP”.^50^ Some surgeons will quote a BP level as a cut-off beyond which they will not operate, though others will consider all factors when deciding whether or not to proceed with surgery. There are no good studies that have looked at the effect of acutely lowering BP on surgical risk, and the exact mechanism by which choroidal hemorrhage occurs is still not known.^30,31^ It is possible that BP measurements are in fact a surrogate measure of the fragility of choroidal vessels (due to previously uncontrolled BP), and, therefore, an acute reduction of BP may not be helpful. Efforts to urgently reduce an elevated BP may occasionally cause a life-threatening systemic hypotension, or other serious problem. The 2012 National Guideline on Local Anesthesia for Ophthalmic Surgery stated: “Uncontrolled hypertension may increase the risks of systemic and ophthalmic complications. However, there is insufficient evidence to support a specific value above which surgery should be deferred. Patients who are taking anti-hypertensive medication should continue their drugs up to and including the day of surgery. Rapidly lowering BP immediately prior to surgery is not advised”.^6^ Thus, it is a good idea to check the patient’s BP soon after (or before) a decision to go ahead with trabeculectomy, so that any hypertension can be treated prior to the day of surgery.

During trabeculectomy, further surgical steps can be taken to minimize the risk of choroidal hemorrhage. Options include gradual ocular decompression via a paracentesis, and maintaining IOP with intracameral viscoelastic or by using an anterior chamber maintainer, and using multiple/tighter sutures on the flap. For some high-risk cases, particularly those that suffered a choroidal hemorrhage in the first eye, it might be preferable to choose an alternative approach, e.g., non-penetrating surgery, or cycloablation.

### Surgical Complication: Decompression Retinopathy

Decompression retinopathy has a clinical appearance similar to that of a retinal vein occlusion. It is occasionally seen after trabeculectomy, and can cause transient or permanent visual loss. The condition is thought to occur in response to sudden lowering of IOP, particularly if the IOP was high prior to surgery.^51^ Thus, much of the above discussion on choroidal hemorrhage may also apply to decompression retinopathy. Again, there is little actual evidence in the literature, though one retrospective study found no difference in incidence when comparing subconjunctival LA and GA.^52^

### Surgical Outcome: Anesthesia Technique and Long-term IOP Control

In the 1990’s, the National Audit of Trabeculectomy (an observational cohort study) suggested that subconjunctival LA might have a higher rate of trabeculectomy failure, when compared to other LA techniques.^53^ However, this study only had a small number of cases that used subconjunctival LA, and this finding has not been replicated. Other studies have found no difference in outcome, when comparing subconjunctival LA and Ga^54,55^ and subconjunctival *vs* Topical,^58^ or subconjuctival *vs* retrobulbar (non-penetrating surgery).^56^ A recent review article on ‘modern filtration surgery’ concluded that ‘subconjunctival anesthesia is safe’.^59^

Several prospective randomized studies have looked at IOP control and anesthesia techniques. Unfortunately, all are relatively small and underpowered, as they tend to be extensions of studies that looked primarily at patient satisfaction. Most reported IOP at 1 year. These prospective randomized trials have failed to show any difference in trabeculectomy success rate when comparing―subconjunctival *vs* retrobulbar (non-penetrating surgery),^56^ subconjunctival *vs* topical,^58^ or peribulbar *vs* topical (phacotrabeculectomy).^57^ Current evidence indicates no major effect of anesthesia technique on the level of IOP after trabeculectomy.^2^

### Surgical Outcome: Postoperative Complications

Previous studies have raised some concerns regarding late postoperative complications and/or bleb failure. One retrospective study compared two cohorts of patients who had had either GA or subconjunctival LA (2% lidocaine) for trabeculectomy. The subconjunctival LA group had a higher incidence of thin-walled, leaky blebs.^60^ It is likely that other factors caused this difference, because other studies, including the author’s own surgical audit using 0.5% lidocaine^1^ have not shown any excess of leaky bleb after subconjunctival anesthesia. There are some evidences to suggest that lidocaine may be inhibitory to conjunctival fibroblasts, with possible beneficial effects akin to the antimetabolites, though this effect does not appear to be strong enough to impact significantly on IOP control or bleb leakage.^1^ Thus, current evidence indicates that anesthesia technique has no major effect on postoperative complication rate.^2,59^

### Special LA Techniques for Trabeculectomy: Topical-subconjunctival-Intracameral Anesthesia

Any LA or GA technique can be used for trabeculectomy, as discussed above. These techniques are described in detail elsewhere.^9^ Some surgeons use minimal anesthesia for trabeculectomy, e.g., topical cocaine 10%,^61^ topical lidocaine gel,^62^ or topical tetracaine applied on a sponge for 5 minutes preoperatively.^63^ Others may commence surgery under topical anesthesia, then give a sub-Tenon’s LA, via the trabeculectomy incision.^64^ This may be combined with intracameral anesthesia.^65^

This author’s preferred technique is topical-subconj unctival-intracameral LA, usually without sedation.^1,2^ This technique has been used for over 12 years for virtually all of my LA trabeculectomies. I use this because it has none of the risks that are associated with sharp-needle periocular injections, and the anterior LA means that the risk of “wipe-out” and other complications is minimized. The technique is quick and cheap, and is easily administered by the surgeon using readily-available equipment and medication. The technique gives good operating conditions, good patient satisfaction, and fears about excess bleb failure or leakage have proved unfounded.^1,59^ It can be used for primary or revision trabeculectomies, tube surgery, and is also suitable for trainee surgeons.

**Fig. 1:**
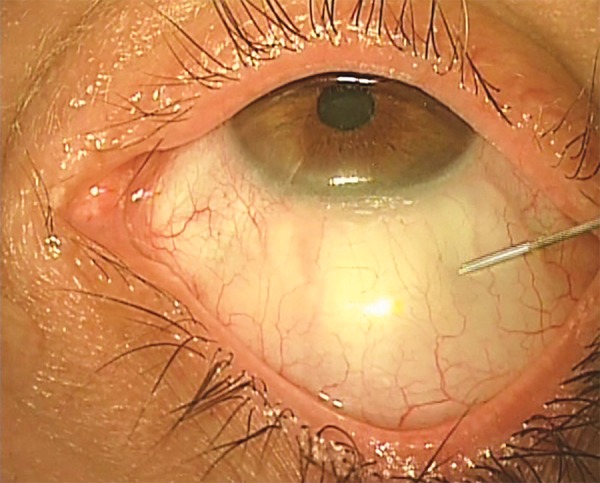
Topical-subconjunctival-intracameral anesthesia for trabeculectomy. Initial injection of 0.5 ml of 0.5% lidocaine, via a fine needle (e.g., 27G), to cover the area of the proposed trabeculectomy bleb (surgeon’s view of a right eye: patient is directed to look downward)

**Fig. 2:**
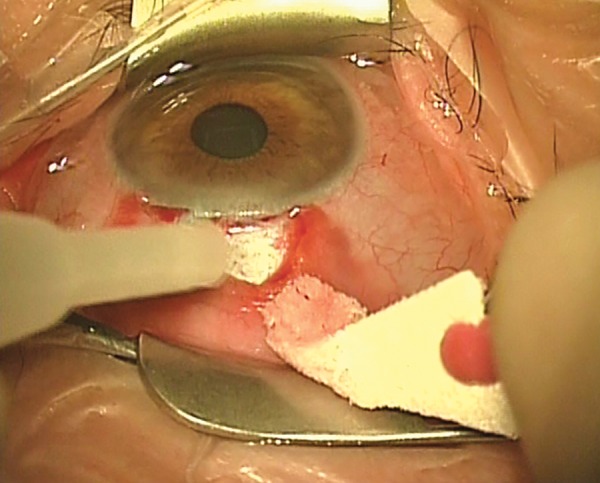
Topical-subconjunctival-intracameral anesthesia for trabeculectomy. Non-preserved tetracaine is placed on to bare sclera, prior to diathermy

**Fig. 3:**
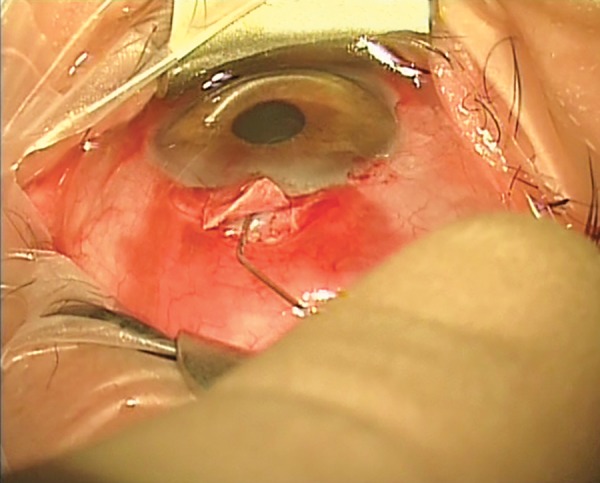
Topical-subconjunctival-intracameral anesthesia for trabe-culectomy. Intracameral non-preserved lidocaine 0.5% is irrigated into the anterior chamber, prior to iridectomy

A suggested method of administering topical-subconjunctival-intracameral LA is as follows. After instilling a topical anesthetic, non-preserved lidocaine 0.5% is injected subconjunctivally via a fine needle (Fig. 1). The initial injection of about 0.5 ml covers the area of the proposed trabeculectomy bleb, and is made under the operating microscope to avoid blood vessels and minimize the risk of hemorrhage. I prefer to give the subconjunctival LA prior to scrubbing and gowning, as this allows a few minutes for the anesthetic to work; this probably gives better analgesia. However, it is possible to give the subconjunctival LA at commencement of surgery. Additional topical non-preserved tetracaine 1% is applied to bare sclera prior to diathermy (Fig. 2). Before an iridectomy, intracameral lidocaine 0.5% (non-preserved) is instilled via a paracentesis, or through the trabeculectomy ostium (Fig. 3). This technique allows for efficient use of resources and gives good patient satisfaction ratings, though occasionally it is necessary to instil additional tetracaine for conjunctival suturing. The LA can be topped up with additional subconjunctival LA, or sub-Tenon LA (via the surgical wound), if needed. Audit has shown good patient satisfaction, excellent IOP control and no excess of bleb leakage with this technique.

**Fig. 4:**
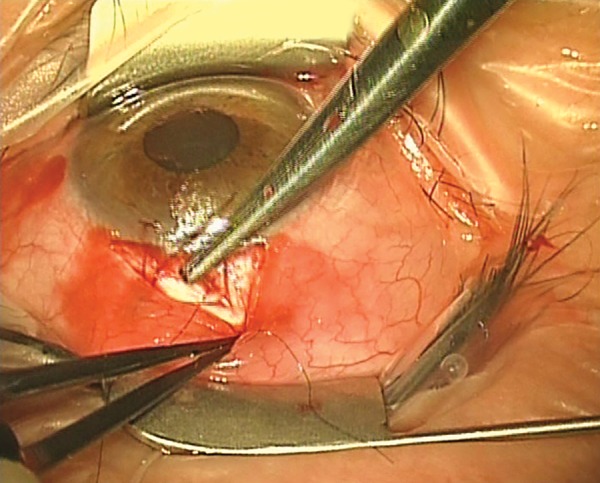
Topical-subconjunctival-intracameral anesthesia for trabeculectomy. This technique means that a corneal stay suture is not needed, making surgery quicker and easier

Topical-subconjunctival-intracameral LA has the additional advantage that a stay suture is not needed. Because eye movements are unaffected by this LA, eye movement can be used to the surgeon’s advantage. The patient is asked to look downwards. This means that surgery is quicker, and the absence of a corneal traction also makes it easier to get the tension right when suturing (Fig. 4). The lack of stay suture does necessitate a degree of co-operation from the patient, who must be able to follow the instruction “look downward”. Therefore, patients who are deaf or who cannot understand the surgeon’s language may need a corneal stay suture. The author can remember only one patient who was unable to keep their eye still with this technique. The problem was easily overcome by administering a posterior sub-Tenon’s LA. This was done “on the table” by the surgeon, via an inferonasal incision, after which a corneal stay suture rendered the eye immobile. In common with all prolonged procedures under LA, patients appreciate the opportunity to wriggle/reposition themselves more comfortably, every 10 minutes or so.

## KEYPOINTS

- A complication of local or general anesthesia might potentially cause blindness or even death. Glaucoma patients may be at higher risk of some complications.
- Posterior placement of LA may cause “wipe-out”, other sight-threatening complications, and life-threatening complications.
- Choose the right anesthetic for the patient
- Choose the right anesthetic for the eye
- Guidelines are available for local anesthesia in ophthalmic surgery
- All anesthesia techniques may be used. The author recommends subconjunctival-intracameral LA for most patients, because of low risk of anesthetic complications or wipe-out, quick and easy surgery, good patient comfort, and good IOP outcome
- General health should be optimized prior to surgery (e.g. diabetes, hypertension controlled)
- Consider whether anticoagulant or aspirin should be stopped around the time of surgery
- Consider whether eye drop/preservative load should be reduced prior to surgery, to maximize chance of success
- Consider whether preoperative anti-inflammatory drops might to maximize chance of success
- Preoperative povidone-iodine drops will reduce the risk of postoperative endophthalmitis.
